# CF2 Represses *Actin 88F* Gene Expression and Maintains Filament Balance during Indirect Flight Muscle Development in *Drosophila*

**DOI:** 10.1371/journal.pone.0010713

**Published:** 2010-05-25

**Authors:** Kathleen M. Gajewski, Robert A. Schulz

**Affiliations:** 1 Department of Systems Biology, University of Texas M. D. Anderson Cancer Center, Houston, Texas, United States of America; 2 Department of Biological Sciences, University of Notre Dame, Notre Dame, Indiana, United States of America; University of Missouri-Kansas City, United States of America

## Abstract

The zinc finger protein CF2 is a characterized activator of muscle structural genes in the body wall muscles of the *Drosophila* larva. To investigate the function of CF2 in the indirect flight muscle (IFM), we examined the phenotypes of flies bearing five homozygous viable mutations. The gross structure of the IFM was not affected, but the stronger hypomorphic alleles caused an increase of up to 1.5X in the diameter of the myofibrils. This size increase did not cause any disruption of the hexameric arrangement of thick and thin filaments. RT-PCR analysis revealed an increase in the transcription of several structural genes. Ectopic overexpression of CF2 in the developing IFM disrupts muscle formation. While our results indicate a role for CF2 as a direct negative regulator of the thin filament protein gene *Actin 88F* (*Act88F*), effects on levels of transcripts of *myosin heavy chain* (*mhc*) appear to be indirect. This role is in direct contrast to that described in the larval muscles, where CF2 activates structural gene expression. The variation in myofibril phenotypes of CF2 mutants suggest the CF2 may have separate functions in fine-tuning expression of structural genes to insure proper filament stoichiometry, and monitoring and/or controlling the final myofibril size.

## Introduction

The indirect flight muscles (IFM) *of Drosophila* are exquisitely adapted to provide the maximum power to the wings. They contain unique isoforms of structural proteins such as actin, the troponins, and myosin heavy chain (mhc). Thick and thin filaments are arranged in a distinctive hexagonal pattern (with each thick filament surrounded by six thin filaments) that allows for maximum contact between actin and myosin molecules. A number of mutations in structural genes are known that allow relatively normal development and functioning of other muscle types, but severely disrupt IFM development and function. Some of these mutations affect splicing of an IFM specific exon (for example the *hdp* [*3*] mutation of *troponin I* (*Tn I*) [1]) or coding regions of genes unique to the IFM (such as *actin 88F* (*Act88F*)), but some are null or strong hypomorphic mutants of genes that are expressed in all muscle types. For example, flies heterozygous for the *Mhc* [*1*] null allele cannot fly, although heterozygous larvae have no discernable locomotion phenotype and heterozygous adults can walk [2]. Likewise, flies lacking a copy of *Act88F* are flightless. Interestingly, flies that are doubly heterozygous for null mutations in *mhc* and *Act 88F* (therefore having a 1∶1 mhc: actin ratio) can fly, although not as well as wild type (with a 2∶2 ratio) [3]. Therefore, it is not just the amounts of these proteins, but also their stoichiometry, that influences flight muscle structure and function.

Sarcomeres assemble from the center outwards in the pupal myotubes. The first thick and thin filaments can be detected at about 42 hours after pupa formation (APF). Additional filaments are added at the periphery until the myofibril reaches its final size of approximately 35 thick filaments across (∼1.5 µm in diameter) in the late pupal stages [4]. Observations of myofibril assembly in *Act88F* null mutants [3] or *mhc* null mutants [2] show that thin or thick filaments can assemble in the absence of the other. Z-discs can still form without the presence of thick filaments, and M-lines can be observed if thin filaments are missing. But the presence of both is required for normal sarcomere size, order, periodicity, and consequentially function [3]. When filament stoichiometry is altered, the peripheral regions of the myofibril tend to be the most severely affected. When there is only one functional copy of *mhc*, the thick filaments at the edges of the myofibril are surrounded by 9–10 thin filaments, instead of the normal six (which is maintained in the central portions) [2]. Extra doses of *mhc* (the equivalent of four copies) result in excess thick filaments at the periphery [5]. Since filament stoichiometry is so crucial to IFM development, it should be very tightly controlled, but little is known about the mechanisms that sense and adjust thick: thin filament ratios.

One point of control is the transcription of muscle structural genes, and the actions of various transcription factors, particularity in the embryonic stages, have been characterized. The MADS box protein Mef2 is a major player in muscle differentiation. Binding of Mef2 is essential for expression of structural genes such as *mhc*, *myosin alkali light chain*, and *myosin light chain 2* in the embryonic dorsal vessel [6], *paramyosin/miniparamyosin* in various muscles in larvae and adults [7], *Tn I* in adult and embryonic muscle [8], and *tropomyosin 1* and *57B actin* in embryonic muscles [9], [10]. *Chorion factor 2* (*CF2*) encodes a Zn finger transcription factor, which was first characterized as a repressor of dorsal fate in the follicle cells of the ovary [11]. It is also expressed in the nuclei of all muscle types of the embryo in a pattern similar to Mef2, being detectable at around stage 12, after the induction of Mef2 expression [12]. CF2 was the first collaborating factor for Mef2 to be characterized [13]. The combination of Mef2 and CF2 has a synergistic effect on the transcription of *57B actin*, *Tn I*, and *mhc* in embryonic muscles [13], and there are clusters of Mef2 and CF2 binding sites upstream of *troponin T*, *tropomyosin 1* and *2*, and *paramyosin* [14]. But the role of CF2 in development of the IFM remains undefined. Previous work [14] reported impaired flight in two hypomorphic *CF2* mutants, but there have been no studies investigating what type of role *CF2* plays in IFM development.

In this paper we investigate the regulatory role of the Zn finger transcription factor CF2 in the development of the IFM. We show that the specific isoforms expressed in the IFM changes during the process of development, with pupal IFM expressing a different set of CF2 isoforms than adult IFM. In contrast to its reported role in embryonic and larval muscles, our data point to CF2 as a repressor, rather than an enhancer, of at least one IFM structural gene, *Act88F*. A reduction in CF2 function increases transcription of several structural genes and in more severe cases increases myofibril size. Gain of CF2 function has a deleterious effect on IFM development, resulting in greatly reduced or almost completely ablated muscles. Similar to its role in embryonic muscle, CF2 in the IFM may be needed for the fine-tuning of structural gene expression. Our data also suggests a role for CF2 in insuring correct myofibril size.

## Results

### CF2 is expressed in adult and developing pupal flight muscle

**Figure 1:**
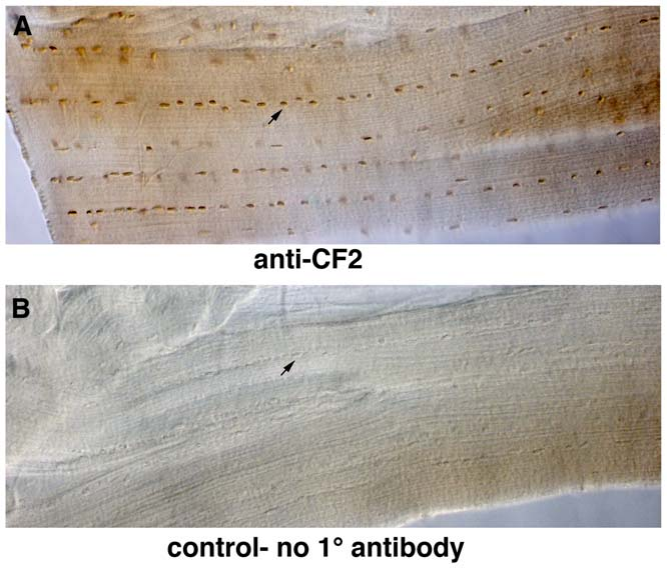
CF2 exhibits nuclear localization in adult indirect flight muscles. A) Flight muscles of a wild type adult, stained with anti-CF2 antibody. B) Control staining without primary antibody. Arrowheads indicate a representative nucleus in each panel.

In the *Drosophila* embryo the CF2 protein is expressed in the nuclei of all three muscle types (somatic, visceral, and dorsal vessel) [12]. To determine whether the protein is also expressed in adult flight muscle, we immunostained dissected IFM with a polyclonal CF2 antibody (that detects all isoforms). As in the embryo, the stain is distinctly nuclear (Fig. 1A), and its specificity confirmed by a control with 2° antibody, but no 1° antibody (Fig. 1B). This nuclear expression pattern is consistent with the conclusion that CF2 is active in the IFM.

**Figure 2:**
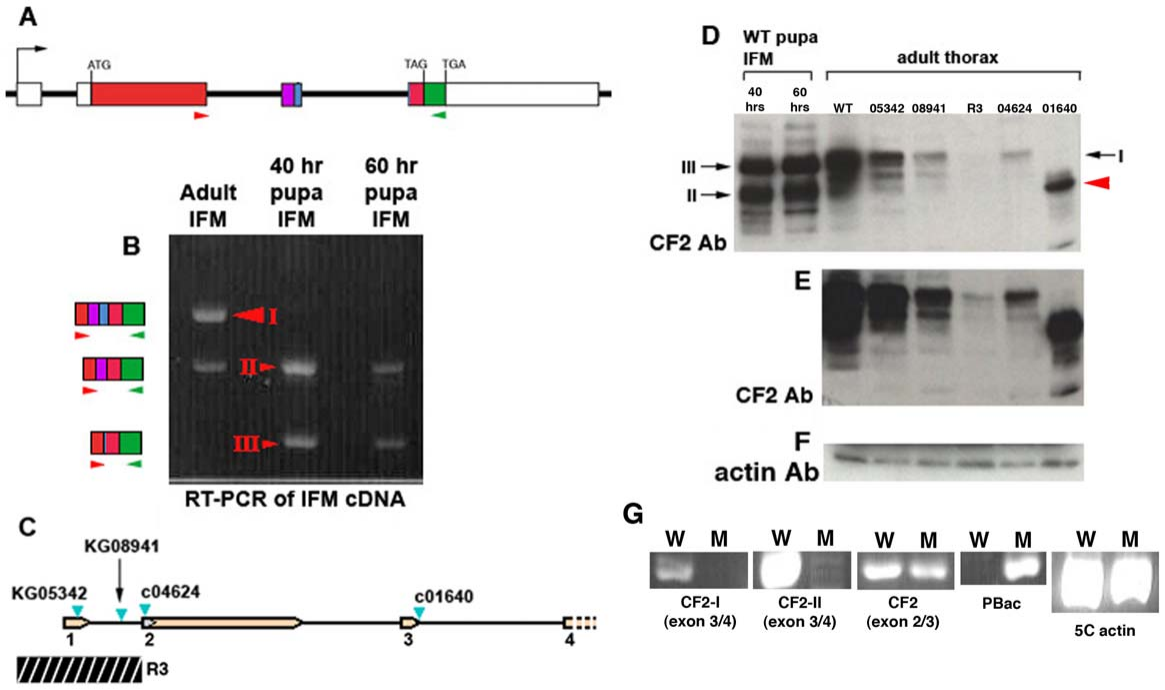
Expression patterns of CF2 isoforms in the IFM. A) Map of the CF2 gene, showing the location of the primers used for RT-PCR (red and green arrows). These primers will amplify all three CF2 isoforms. Red regions denote coding sequences that are common to all three isoforms, purple common to isoforms I and II, blue unique to isoform I, and green unique to isoform III. The lack of exon 3 in isoform III causes a frame shift in exon 4, so that the green colored region, while present in all three isoforms, is in frame only for isoform 3. B) Gel of RT-PCR reactions with IFM cDNA from adults, ∼40 hr pupae, and ∼60 hr pupae. The predicted exon content of each band is indicated by the colored boxes on the left. All PCR products were sequenced to confirm their exon content. C) Diagram of the *CF2* gene and the sites of P-element insertions or genomic DNA deletion. The hatched bar denotes the region that contains the *R3* deletion. The exact breakpoints are unknown, but PCR mapping confirms that it is upstream of the ATG site. D) Western blot of IFM protein from wild-type pupae and whole thorax protein from wild type and mutant adults, using a CF2 antibody. The red arrow head points to the altered form produced by the *01640* mutant. E) Longer exposure of D), to show the fainter bands. F) Loading control Western using an actin antibody. G) RT-PCR tests on mutant *01640 CF2* transcripts. Primers specific for isoforms I and II are 3′ to the *01640* insertion site (See panel C), from exons 3 and 4. A primer set 5′ to the *01640* insertion site (from exons 2 and 3) was used to verify transcription of the CF2 gene. Testing for the presence of P-element sequences used a primer from exon 2 and the PBac-3F2 primer [17]. 5C actin primers served as a loading control.

To examine the expression of CF2 transcript during development of the flight muscles, we used RT-PCR on cDNA from the dissected IFM of adults, 40 hr APF pupae (beginning of myofibrilogenesis), and 60 hr APF pupae (elongation of IFM complete). The primers used were designed to amplify all three reported CF2 isoforms (Fig. 2A), which are created by alternative splicing of exon 3 [15], [16]. In the adult sample (Fig. 2B), only isoforms I and II are detected. However, in both pupal lanes, isoform III replaced isoform I. This result is confirmed by Western blots (Fig. 2D–E). In the pupal samples the predominant bands run at 53.5 kD and 56.3 kD, the expected sizes for isoforms II and III. This suggests a previously unknown role for isoform III, which had been reported to be only expressed in testes [15]. In the wild type adult sample, a band the size of isoform I (∼56.7 kD) is observed instead of isoform III, consistent with the PCR results.

We obtained four P-insertion mutant alleles of *CF2* (diagrammed in Fig. 2C) (*CF2[KG05342]*, *CF2[KG08941, CF2[c04624]* and *CF2[c01640]*, in fly lines referred to hereafter as *05342, 08941, 04624, and 01640*). *05342, 08941*, and *04624* have P-insertions 5′ to the ATG start site in exon 2. The P-element in the *01640* line is in exon 3, within sequences unique to isoform I. We generated a fifth mutant (*CF2[KG08941-R3]* ), referred to hereafter as *R3*, by excision of the P-element from line *08941*. The *R3* mutant has a small deletion (∼2.7 kb) that removes the first exon of *CF2*, but leaves the second exon (with the ATG start site) intact. All mutants are homozygous viable, and exhibit no visible signs of IFM defects, such as abnormal wing position. Western blots of mutant adult thoraces reveal that four of the five mutations are hypomorphic, ranging from the least severe, *05342*, to the most severe, *R3*, which has a greatly reduced level of all forms of CF2 as compared to wild type (Fig. 2D–E).

The fifth mutant, *01640*, has no detectable levels of isoform I. There is one very strong band, but it does not match the sizes for any of the other CF2 isoforms found in the other lanes. It runs higher than isoform II, but lower than isoforms I and III. *01640* is unique among the set of mutants in that it is the only one to have a P-element insertion within coding sequences, in the region of exon 3 that is found only in isoform I. To test the possibility that the *01640* mutation produced an aberrant protein, we did RT-PCR with several sets of primers designed to test the splicing of exon 3, all CF2 isoforms, or the presence of P-element sequences (Fig. 2G). With primers sets specific for isoform I or II (and 5′ to the P insertion site), the expected PCR products (386 bp and 361 bp) are not observed in the mutant cDNA lanes, in contrast with the wild type cDNA lanes. Primers from exons 2 and 3 upstream of the P-site produce identical results (a 228 bp band) with both genotypes, confirming that CF2 transcription is initiated in *01640* mutants. As expected, when primers for CF2 exon2 and the PBac{PB} P-element vector [17] are used, a 328 bp PCR product is found in the *01640* lane, but not the wild type lane. These results confirm that exon 3 is not properly spliced in *01640* mutants, the mRNA contains P-element sequences, and this likely produces a truncated form of the CF2 protein.

### Loss of CF2 function causes subtle defects in IFM structure

**Figure 3:**
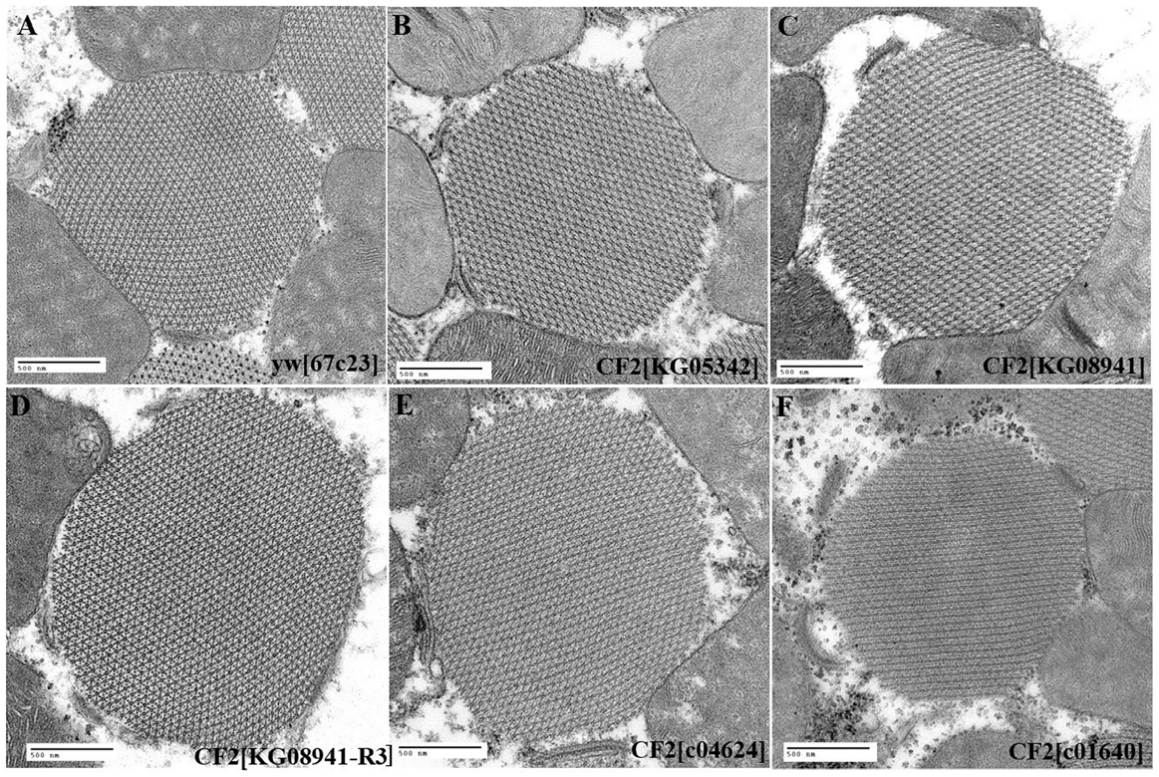
Myofibril phenotypes of CF2 mutants. All pictures are cross sections through IFM tissue, with a magnification of 60,000X. A) *y w[67c23]* B) *CF2[KG05342]* C) *CF2[KG08941]* D) *CF2[KG08941-R3]* E) *CF2[c04624]* F) *CF2[c01640]*. Scale bars are 500 nm.

The IFM of CF2 mutant adult flies appear normal on the gross structural level, with no abnormalities in muscle number, size, or patterning observed (data not shown). However, at the ultrastructural level, differences in myofibrils are seen between the wild type and mutant flies (Fig. 3). In *05342*, *08941*, and *01640* myofibrils (Figs. 3B, C, and F), the filament pattern often appeared diffuse. The round shape of the myofibrils indicates a proper cross section cut (as opposed to a section at an angle, which would produce an oval shape to the myofibril), and filament patterns of simultaneously prepared wild type samples were clearly in focus (Fig. 3A). In contrast, all mutants had clearly discernable sarcomeres in longitudinal sections (data nor shown). *R3* filaments were always sharp and well defined, and these myofibrils showed no abnormalities in filament pattern. Most *04624* filaments were also easily visualized with a normal filament arrangement, although they tended to be slightly less sharp than wild type or *R3*.

Myofibrils from CF2 mutants *08941*, *04624*, and *R3* (Figs. 3C, D, and E) are clearly larger than the wild type control (Fig. 3A). We quantified the changes in myofibril size by counting thick filaments in wild type, *R3* and *04624* myofibrils, as these measurements are less prone to any distortion during fixation and cutting of the sections. A count of thick filaments from 13 randomly selected myofibrils from wild type, R3, and 04624 confirms this observation. Wild type myofibrils contained an average of 802.9±34.5 thick filaments, whereas *04624* and *R3* averaged 1215.0±120.6 (df = 24, t = 12.08, P<0.0005) and 1316.3±64.1 (df = 24, t = 25.43, P<0.0005), respectively, a statistically significant increase in myofibril size. Therefore a certain threshold of CF2 function appears to be required to insure the proper myofibril size.

### CF2 loss of function increases the levels of mRNAs encoding structural proteins

**Figure 4:**
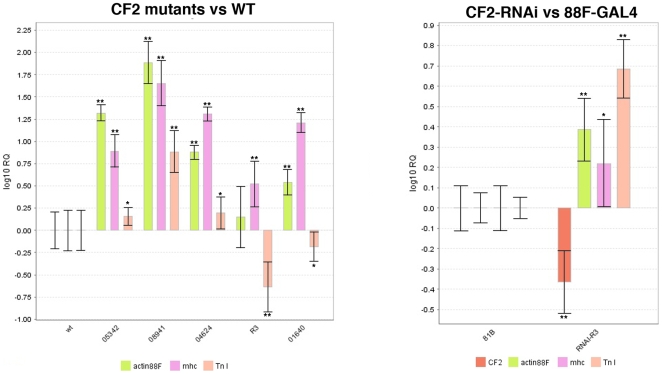
Transcription of muscle structural genes is altered in *CF2* loss of function mutants. Graphs of a representative qPCR run from A) Five different CF2 mutant lines tested against a wild type strain, B) *88F-GAL4[81B-13]/+;11924-R3 CF2-RNAi/+* tested against *88F-GAL4[81B-13]/SM6*. ** denotes differences from the control with a P-value ≤0.001. * denotes a P-value <0.05.

To determine the mechanism for the observed ultrastructural phenotypes in some CF2 mutants, we examined expression of several IFM structural genes via RT-PCR, using whole thoraces (minus wings and legs). These results indicated elevations in mRNA levels for most of the CF2 mutants (data not shown). To verify and quantitate these results, we next performed qPCR on cDNA from the five CF2 mutants and a wild type control, using primers for *Act88F*, and the flight muscle specific isoforms of *mhc* and *Tn I.* The results of the most consistent qPCR run are diagrammed in a graph in Fig. 4A. The *08941* mutant showed the most dramatic effects, averaging (over 2 separate runs) a 71-fold increase in *Act88F* RNA levels, a 50-fold increase for *mhc*, and a 8-fold increase for *Tn I*. The *05342* and *04624* mutants were the next strongest by this measure. *05342* had average transcript elevations of 18x, 7x, and 1.3x for Act88F, *mhc*, and *Tn I*; for *04624* the same RNA levels were up 7x, 20x, and 1.5x. The *01640* mutant had a weaker RNA phenotype, with *Act88F* up an average ∼3.5x, *mhc* up 14x, and *Tn I* down 1.6x. The *R3* transcription phenotype was the mildest, with only a 1.7-fold increase in *Act88F*, a 3.7x increase for *mhc*, and a 3.6-fold decrease in Tn I. These results were subjected to student's t-test to verify statistical significance, and with the exception of one of the *R3 act88F* assays, all P values were 0.05 or lower. Quantitative PCR testing of *CF2* transcript levels in these mutants showed no significant changes between the mutants and wild type, (data not shown).

To further verify that these effects are due to loss of CF2 function, we repeated this experiment with two *CF2-RNAi* lines, expressed via an *88F-GAL4* driver (Figure 4B). CF2 specific primers (which detect all isoforms) show that the RNAi construct does indeed lower *CF2* transcript levels, by over 50% *11924-R2* line, compared to the driver only control. The *11924-R3* line increased *Act88F* transcript levels almost 2.5x, *mhc* over 1.5x, and *Tn I* by almost 5 fold. These differences are not as great as those observed between the stronger *CF2* mutants and wild type, but the *88F-GAL4* has its own effects of muscle structural genes. Compared to wild type, the driver alone caused elevations in mRNA levels of the three structural genes tested, in addition to *CF2 its*elf (data not shown). Addition of a UAS-CF2-RNAi construct reduced the levels of *CF2* mRNA, but caused further increases in *Act88F, mhc*, and *Tn I* transcripts, consistent with a reduction of CF2 function increasing levels of structural gene mRNAs. Taken together, analysis of six fly lines with altered CF2 function demonstrates that CF2 represses expression of these muscle genes in the IFM.

### CF2 overexpression disrupts IFM development

**Figure 5:**
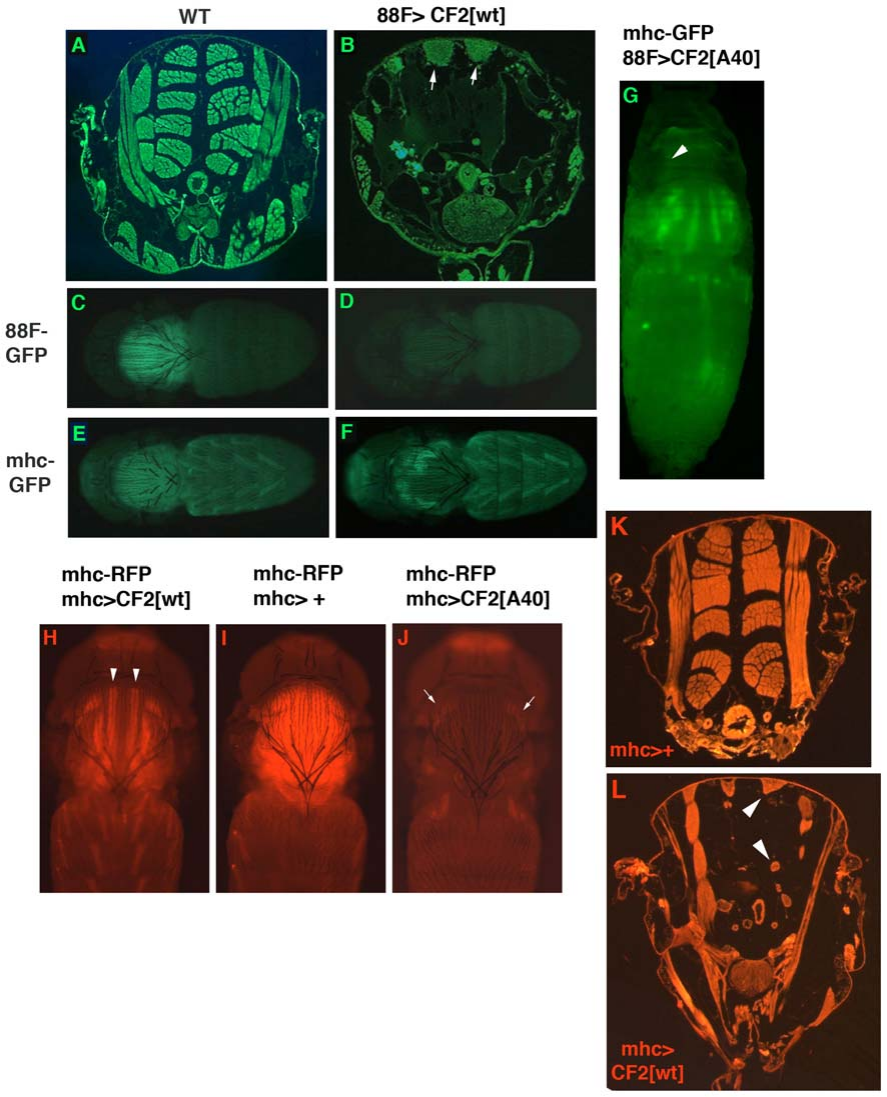
CF2 gain of function disrupts IFM development. A) Cross section of the thorax of an *88F-GAL4[81B-13]/CyO* fly (wild type control). B) Cross section of the thorax of an *88F-GAL4[65-9]/UAS-CF2-II[wt])* fly. White arrows point to the remnants of the two dorsal-most dorsal longitudinal muscles C) Expression of an *88F-GFP* marker in wild type IFM (genotype *88F-GAL4[81B-13]/CyO*). D) Expression of an *88F-GFP* marker in IFM of fly overexpressing wild type CF2 (genotype *88F-GAL4 [81B-13]/+; UAS-CF2-II[wt]*). E) Expression of a *mhc-GFP* marker in wild type IFM (genotype *88F-GAL4[65-9]/TM3, Sb*). F) Expression of a *mhc-GFP* marker in IFM of fly overexpressing wild type CF2 (Genotype *88F-GAL4[65-9]/UAS- CF2-II[wt]*). G) Defects in muscle development in a *88F-GAL4[65-9]/UAS-CF2-II[A40]* pupa. The age can be approximated by the presence of pigment in the eye (indicated by the arrowhead. H) Pharate adult expressing *UAS-CF2[wt]* via a *mhc-GAL4* driver. Arrowheads point to thinner IFMs. I) Control *mhc-GAL4, mhc-RFP* animal, at the same stage as H and J. J) Pharate adult expressing *UAS-CF2[A40]* via a *mhc-GAL4* driver. Arrows indicate the anterior remnants of the IFM. K) Section through the thorax of a control fly expressing the *mhc-GAL4* driver. L) Thoracic section of a fly expressing UAS-CF2-II[wt] via the *mhc-GAL4* driver. Arrowheads point to two of the abnormally thin dorso-lateral IFM.

If CF2 has a role as a repressor in the IFM, then an increase in function should cause a decrease in transcript levels of genes encoding structural proteins. To test this hypothesis we overexpressed wild type and mutant (T40A) forms of CF2 using an IFM specific GAL4 driver containing 1.3 kb of DNA upstream of *Act88F*. This driver mimics the IFM expression of Act88F, with expression first detected at around 40 hours APF. Ectopic CF2 expression caused major disruption of the IFM. Fig. 5A shows a cross section through a wild type thorax, with its characteristic indirect flight muscle pattern. When wild type CF2 is overexpressed (Fig. 5B), only a few shreds of muscle tissue can be found at the dorsal part of the thorax; the majority of the space is empty of any muscle tissue. The T40A mutant form (UAS-CF2[A40]), which removes a phosphorylation site crucial to control of the subcellular localization of CF2 (via nuclear export) [18] has an even more severe effect (data not shown).

**Figure 6:**
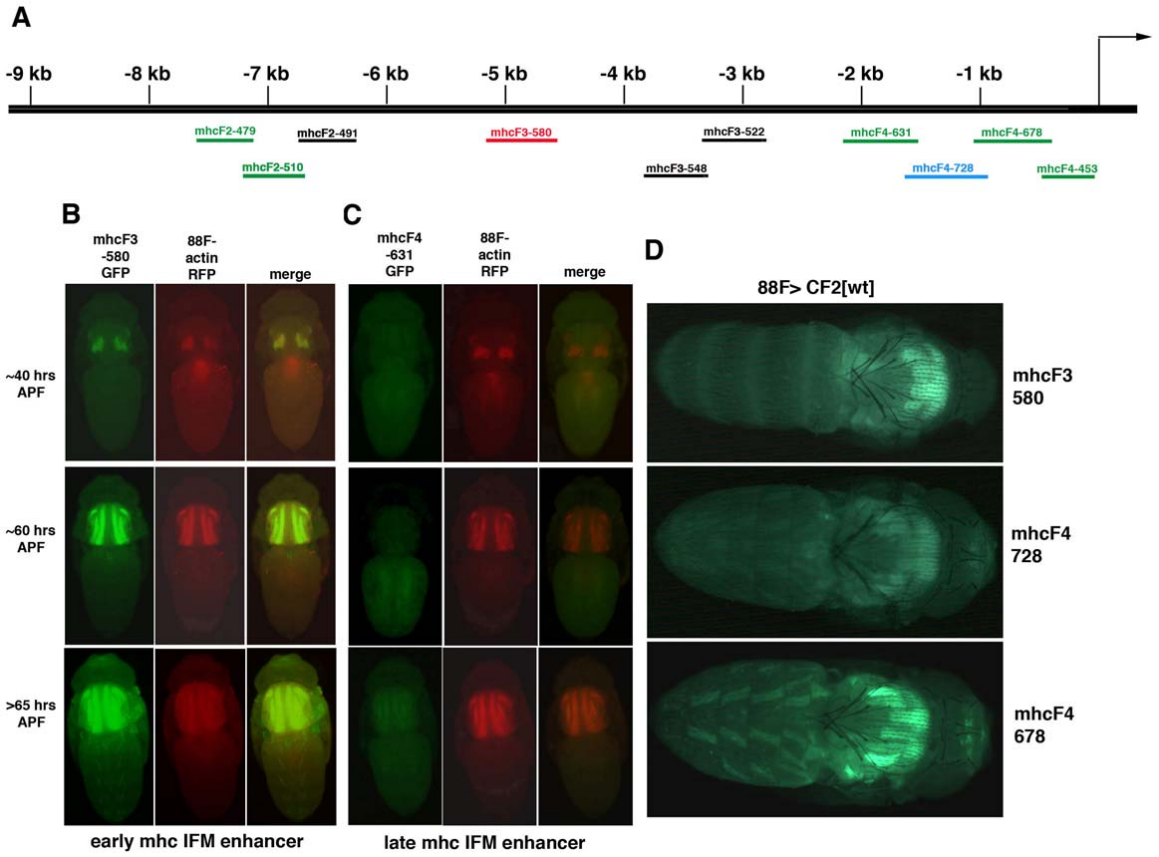
CF2 overexpression does not directly inhibit *mhc* transcription in the IFM. A) Map of the upstream region of *mhc*, showing regions that drive muscle expression. Green bars (late IFM elements) denote elements that express later in IFM development (after ∼70 hrs APF, class 3), red indicates an element (early IFM element) that is expressed at the onset of myofibrilogenesis (∼40 hrs AFP at 22°C, class I), and blue an early IFM element that ceases expression in early adulthood (class 2). The black bars denote enhancer elements that drive expression in muscle types other than IFM. B) Expression patterns of GFP and RFP in animals carrying both the *mhcF3-580-GFP* early enhancer and an 88F-RFP reporter. C) Expression patterns of GFP and RFP in animals carrying both the *mhcF4-631-GFP* late enhancer and an 88F-RFP reporter. D) Expression of the three different classes of *mhc* reporters in a CF2 gain of function background.

The use of two different GFP reporters produced two strikingly different results. When an Act 88FGFP reporter was introduced into these genetic backgrounds, there is no expression of GFP in the CF2 gain of function, even in the remnants of muscle tissue (Fig. 5D, wild type control Fig. 5C). In contrast, a *mhc-GFP* marker is not affected. GFP expression is strong in the muscle remnants (Fig. 5F, wild type control Fig. 5E), which are concentrated in the dorsal and anterior most portion of the thorax. Examination of developing IFM in pupa with overexpressed CF2[A40] and a *mhc-GFP* marker revealed that much of the muscle tissue fails to develop (Fig. 5G). The animal had reached to stage where its eyes were pigmented, which makes it further along in its development than the animals shown in the bottom panels of Figs. 5B and C, yet those younger animals have a full set of IFM close to their final size. We also tested a *mhc-GAL4* driver (constructed with a 580 bp IFM specific enhancer region upstream of *mhc*, see Fig. 6A) with the *UAS-CF2* constructs, and looked at the IFM of pharate adults. The wild type CF2 produced a milder phenotype with this driver (Fig 5H), with the IFM thinner than wild type (Fig. 5I), but intact from anterior to posterior. Sections of these mutant thoraces (Fig. 5L) confirmed that the IFM were considerably thinner than driver only control IFM (Fig. 5K). The *A40* mutant construct produced IFM disruption as equally severe as seen with the *88F-GAL4* driver (Fig. 5J), with only a few shreds of IFM expressing RFP at the anterior of the thorax. These results demonstrate that an excess of the CF2 transcription factor has a detrimental effect on the development of IFM. The most likely cause is repression of the transcription of structural genes. This is not currently directly testable due to: 1) a reduction is muscle mass if excess CF2 is expressed during muscle development, and 2) the lack of an inducible system that does not have its own effect on structural gene mRNAs. For example, when the GAL80[ts] system was tried in order to induce excess CF2 after IFM development was complete, the higher temperatures required caused a decrease in IFM structural genes of control flies, making such an experiment impossible to interpret (data not shown).

### CF2 may not directly affect *mhc* transcription

Despite the effects of loss or gain of CF2 function on *mhc* transcripts, expression of a *mhc-GFP* reporter is not affected by overexpression of CF2. Since we did not know the precise location of the sequences driving that reporter, we used a series of upstream *mhc* reporters we had generated to examine more precisely the effects of CF2 on *mhc* transcription in the IFM. We had previously searched the 9 kb region upstream of *mhc*, using a series of overlapping constructs for muscle (in particular IFM) enhancer elements. Fig. 6A diagrams the location of regions that drive muscle expression. Our analysis revealed a great diversity in both spatial and temporal control of *mhc* expression. We found multiple elements for a number of different muscle types (listed in Table 1), and of most interest to us, three different types of IFM enhancers. The first type of element (denoted as Class 1, diagrammed in red in Fig. 6A) is expressed at the onset of myofibrilogenesis, in a pattern reminiscent of the Act 88F GFP reporter (Fig. 6B compares the two expression patterns). Expression in the IFM remains strong throughout the adult life of the animal. A Class 2 element (diagrammed in blue in Fig. 6A) also expresses at the beginning of myofibrilogenesis, but the expression is weaker, and it diminishes to undetectable levels within a few days after eclosion. Class 3 elements (diagrammed in green in Fig. 6A), are not expressed until after the IFMs have finished elongating (Fig. 6C compares this pattern with the Act 88F pattern).

**Table 1:** Muscle expression patterns of upstream *mhc* enhancers.

Element	Location	Muscle Expression Pattern
mhcF2-479	−7600 to −7111	larval somatic muscle, jump muscle (mid pupa-adult), IFM (mid pupal- adult)
mhcF2-510	−7201 to −6692	larval somatic muscle, jump muscle (mid pupa-adult), IFM (mid pupal- adult)
mhcF2-491	−6768 to −6278	subset of larval somatic muscles, leg muscle (pupa-adult)
mhcF3-580	−5165 to −4586	IFM (40 hrs APF- adult), dorsal vessel (midpupa), subset of abdominal muscles and upperleg muscles (pharate adult), proboscis muscles (late pupa)
mhcF3-548	−3851 to −3303	jump muscle, dorsal vessel, leg muscle (pupa)
mhcF3-522	−3360 to −2838	larval somatic muscle, proboscis muscles, leg, jump muscle (adult)
mhcF4-631	−2158 to −1528	IFM (mid pupal- adult), dorsal vessel (midpupa)
mhcF4-728	−6857 to −913	larval somatic muscle, IFM (40 hrs APF-early adult)
mhcF4-678	−1048 to −371	larval somatic muscle, jump muscle (mid pupa-adult), IFM (mid pupal- adult), leg muscles, abdominal muscles
mhcF4-453	− 485 to −33	larval somatic muscle, jump muscle (mid pupa-adult), IFM (mid pupal- adult), leg muscles, abdominal muscles

Location refers to the position of the cloned sequences relative to the *mhc* transcription start site, which is considered to be position 0. Animals were examined at the larval, pupal, and adult stages for GFP expression. All elements are diagrammed in Fig. 6A.

We crossed a representative of each of the 3 types of *mhc* IFM enhancer reporter lines into a CF2 gain of function background. The Class 3 element we chose, F4-678, is also expressed in larval somatic muscle, and has a putative CF2-II binding site (−381 to −372) in close proximity to a putative MEF2 site (−412 to −404). It should be noted that constructs F4-728 and F4-678 are contained within a region that has been previously tested for *mhc* muscle enhancers [14], [19]. All three types of enhancers continued to drive GFP expression even when an excess of CF2 protein is present (Fig. 6D). The muscle mass is clearly reduced, and bunched at the anterior of the thorax, but still strongly expresses GFP. Given the reduction of native *mhc* transcripts seen with ectopic expression of CF2 (Fig. 5H), if direct repression by CF2 were the cause, we would expect to see an effect on at least one of the *mhc* reporters. Our results suggest that a reduction in *mhc* mRNA could be caused by a mechanism other than direct transcriptional repression by CF2.

## Discussion

In this paper we investigate the expression pattern and function of the Zn finger transcription factor CF2 in adult flight muscle development. We find that the isoform expression pattern in the IFM changes over time, with isoforms II and III present during the building of the muscles, and isoforms I and II expressed in the adult muscle. We have also characterized the IFM phenotypes of CF2 mutants. In the loss of function mutants, transcripts of three muscle structural genes (*Act 88F*, *mhc*, and *Tn I*) are increased, and the stronger mutations cause an ∼50% increase in myofibril size. However the hexagonal arrangement of thick and thin filaments is not perturbed. CF2 gain of function severely impairs the development of the IFM, most likely by the downregulation of structural genes. Lastly, our data point to CF2 as a probable direct negative regulator of *Act 88F*, but possibly indirect regulator of *mhc*. The exact nature (direct or indirect) of the effects of CF2 on *Tn I* remains to be resolved.

### Dynamic isoform expression pattern of CF2 in the IFM

During the early to mid stages of pupal development, when the sarcomeres are still assembling, strong expression of isoforms II and III at both the mRNA and protein level is observed in the IFM, while isoform I replaces isoform III in the adult stage. This represents a novel expression pattern for isoform III, which was previously reported to be exclusive to the testes [15]. Our methods (RT-PCR and Western blotting) are more sensitive than the combination of PCR and Southern Blots used in this previous study [15], and in addition we used material from dissected IFM or thoraces rather than whole animals. Isoform III shares the first three N-terminal zinc fingers found in isoforms I and II, but the lack of exon 3 causes a frame shift that eliminates the C-terminal zinc fingers. As zinc fingers 4, 5, 5′, and 6 have been shown to be essential for DNA binding [16], isoform III would be unlikely to bind DNA. However, the common three N-terminal zinc fingers could facilitate protein-protein interactions. A possible function for isoform III is to modulate the activity of isoform II, by sequestering co-factors that bind the N-terminal region. Isoform III could be needed to prevent isoform II from being too active (and thus repressing the *Act 88F* level too much) during myofibrilogenesis. In the adult IFM isoform III is replaced by isoform I. This isoform has an extra DNA binding Zn finger. It preferentially binds a 12 bp consensus site [16], but it too may compete with isoform II, for cofactors if not also for binding sites. Isoform I may be functioning in a “maintenance mode” after the IFM myofibrils have been built to their appropriate size. Earlier studies [14] reported that the 05342 and 08941 mutant lines are flight impaired, and this effect worsened with age. This phenotype could be due to the lowered levels of isoform I in the IFM of these CF2 mutants.

### Reconciling protein/transcript levels and ultrastructural changes in CF2 mutant myofibrils

At first glance there looks to be a number of contradictions between the Western, qPCR, and myofibrilar phenotypes of the five different *CF2* mutants. The Western blot data indicate a decreasing hypomorphic series (based on amounts of CF2 protein) with *R3*>*04624*≥*08941*>*05342*. *01640*, which produces an altered protein, would fall outside this grouping. However, when effects on the amounts of *Act88F* and *mhc* mRNAs are considered, the order of severity is changed, with *08941*>*05342* = *04624*>*01640*>*R3*. The results from measurement of *Tn I* transcripts are more complicated. The *08941*, *05342*, and *04624* mutants all show increased levels of *Tn I* transcripts. The opposite is observed with *01640* and *R3*, which cause a decrease in *Tn I* transcript levels. Expression of *CF2-RNAi* in the IFM causes an increase in *Tn I* mRNA, lending support to the hypothesis that a reduction in CF2 function caused increased transcription of *Tn I* (in addition to *Act88F* and *mhc*). As mentioned above, *01640* is most likely not a simple hypomorphic mutation. Given the presence of P-element sequences in the *CF2* mRNA of this mutant (Fig. 2G), and a band on the Western blot (Figs. 2D and E), that does not correspond to any of the three CF2 isoforms, it is most probable that the *01640* mutation produces a truncated CF2 protein. The *01640* P insertion is within the portion of exon 3 that is uniquely spliced into isoform I. The coding sequences upstream, which include Zn fingers 1-4 (and half of Zn finger 5′) could reasonably be expected to be present in this new CF2 protein. Such a protein could have changed functions, which might account for the differences in effects on muscle gene expression from those seen with the hypomorphic *08941*, *05342*, and *04624* alleles.

Why does *R3* appear to be the strongest CF2 mutant by protein levels, but is the weakest when the effects of muscle gene transcription are tested? The answer may be that it is not just a CF2 hypomorph. *R3* was produced by excision of the *08941* P-element. Preliminary mapping via PCR analysis has indicated that while the ATG site within exon 2 is intact, exon 1 is missing. Furthermore, at least 2.7 kb of DNA upstream of exon 2 is also missing. The nearest gene upstream of CF2, *GC3008*, is only 304 bp upstream of the *CF2* transcription start site. Therefore the *R3* deletion will also affect this gene. *CG3008* is predicted to be a kinase (http://flybase.org/reports/FBgn0031643.html), but there is no current data on its adult expression pattern or phenotypes of any mutants. There are also several uncharacterized genes not far downstream of *CG3008* that could also be potentially affected by the *R3 d*eletion. There is no doubt that *R3* affects *CF2*, given the greatly reduced levels of CF2 protein and the enlarged myofibril phenotype, but changes in any of these neighboring genes could have their own effects on transcription in the IFM. These effects could mitigate some of the effects of reduced CF2 in the *R3* allele. Further mapping of the *R3* deletion and examination of these neighboring genes should clarify this matter. In summary, *08941*, *05342*, and *04624*, which produce increases in muscle gene transcript levels that are in agreement with those produced by RNAi knockdown, are hypomorphic mutations. *01640* is most likely a neomorph, and *R3*, while a *CF2* hypomorph, is also mutant for at least one additional gene.

When considering the ultrastructural changes in *CF2* mutants, one correlation is unambiguous: the three lines with the greatest reductions in CF2 protein (*08941, 04624*, and *R3*) are the ones that display an enlarged myofibril phenotype. With the *R3* and *04624* myofibrils, it is possible to get an accurate measure of the increased size by counting the numbers of thick filaments. *08941* myofibrils appear to be comparable is size, but the lack of any focus on distinct filament structures makes counting thick filaments impossible. There is a weaker correlation here between the lack of sharp filament structures and the increases in structural gene transcription. *08941*, which has the greatest increases in mRNA levels, also has the “fuzziest' myofibrils. The myofibrils of *05342* mutants, which have strong mRNA increases, also lack a clear focus on filament structure. Instead of the hexameric pattern, vague diamond or lines patterns are commonly observed instead. Such patterns can be seen in wild type myofibrils cut at an angle, as opposed to a 90° cross section. However such a cut would create oval, as opposed to round, myofibril slices. Since the slices in our samples are round, indicating a proper cutting angle, we speculate that the filaments in these mutants may not be oriented properly, so that they get cut at an angle. As mentioned, sarcomeric structures are observed in longitudinal section, so obviously thick and thin filaments are present. However there may be subtle defects in how the filaments line up, caused by excess amounts of protein, which could interfere with proper filament alignment. *04624* has an intermediate phenotype; the increases in transcript levels are comparable to *0534*2, but the filament structure begins to come into focus, although it is not as sharply defined as it is in *R3* myofibrils. *01640* does not fit in neatly here, as the transcription phenotype is weaker than *04624*, but the myofibril filament structure is diffuse and does not come into focus.

These differing phenotypes argue against a simple model where an increase in the amount of structural proteins is the direct cause of an increase in myofibril diameter. This suggests an additional function for CF2 that involves sensing/control of myofibril size, in addition to insuring proper filament ratios. Our data show a small but significant increase in myofibril diameter in several of the CF2 mutants over wild type. Even in the case of a mutant such as *R3*, where the filament balance appears to be restored, and the hexameric filament pattern is intact and properly aligned, the increase in myofibril size could be suboptimal for flight, as it could mean less room for mitochondria. Such mutant flies could be capable of short bursts of flight, but lack endurance for longer flights. There is one other report in the literature of a mutant that increases myofibril diameter, *flt H* [20]. These flies are flightless, but there were other defects observed in addition to increased myofibril diameter, such as disorganized filaments and defects in Z-bands, which are not observed in *R3* or *04624* mutants. The gene corresponding to the *flt H* mutation has not been identified, so nothing is known at the molecular level about its role in flight muscle development.

### CF2 functions in adult muscles are not the same as in embryonic muscles

In the embryonic and larval somatic muscles, CF2 plays a role as a transcriptional activator. In cooperation with Mef2, it activates transcription of a number of different structural genes. However, in the developing IFM, our data point to an opposite role. It should be noted that muscle genes that are activated synergistically by Mef2 and CF2 tend to be expressed in both the larval and adult stages [14]. But *Act 88F* is a gene uniquely expressed in the IFM, distinct from the *57B actin* gene expressed in larval muscle. There are putative CF2 binding sites in the first intron of *Act88F*, but no putative Mef2 sites nearby. We see an increase in *Act 88F* mRNAs with CF2 loss of function (hypomorphic mutant background or 88F>CF2-RNAi). We also observe repression of the *Act 88F* reporter. These data point to a likely role for CF2 as a direct transcriptional repressor of *Act88F*. The use of an *88F-GAL4* driver raises a question because it shares most if not all of its sequences with the 88F-GFP reporter: would not the ectopic CF2 negatively feedback and repress its own expression? We suspect that it most likely does, but not before such a massive overexpression of CF2 (as the 88F enhancer is very strong, [21]) does its damage and disrupts IFM development. Because of the potential problems with negative feedback, we also tried a *mhc-GAL4* driver (made from the F3-580 upstream fragment diagrammed in Fig. 6A) to drive ectopic CF2 expression, a reporter that is not quenched by ectopic CF2. The wild type CF2 construct had a milder effect with this driver, resulting in IFM noticeably thinner than wild type, but intact from anterior to posterior, in contrast to the effects of the *88F-GAL4* driver. CF2[A40] behaved the same with both drivers, causing an almost total ablation of muscle tissue. The severity is most likely due to the removal of the phosphorylation site, resulting in a protein that cannot be controlled by shuttling it out of the nucleus [18].

*Tn I* transcript levels are also altered by hypomorphic *CF2* mutations, although we cannot say with our present data whether this effect is direct or indirect. Previous studies [22] demonstrated that the overexpression of *Tn I* caused downregulation of the transcription of other thin filament genes, which implies a system of coordinate regulation of thin filament genes. CF2 could be acting directly on this gene or the effect could be indirect as a response to alterations in level of *Act 88F* transcripts. It has been reported that the *05342* and *08941* mutant lines exhibit a decrease in *Tn I* transcript levels in embryos, pupae, and adults [14], which appears to be in contradiction with our results. The conflicting results may be explained by the differences in how the experiments were performed. We used a primer set from *Tn I* exons 3 and 4 for our qPCR, which would detect only the IFM-specific isoform of *Tn I*. The earlier studies [14] used primers from *Tn I* exons 7 and 8, which would amplify all *Tn I* transcripts. It is possible that a strong downregulation in the non-IFM muscles of the CF2 mutants could mask an increase in *Tn I* transcript in the IFM.

Despite the same kind of effects observed on *mhc* mRNAs as seen with *Act 88F* and *Tn I*, we have no evidence for a direct role for CF2 in the regulation of this gene in the IFM. We tested four different *mhc* reporters (three with known *mhc* enhancer regions) in CF2 gain of function backgrounds, and found no negative effects on GFP expression such as we observed with the *Act88F-GFP* reporter. Intronic *mhc* enhancers have been reported [19] and we have not tested any of these. But the enhancers we did test were strong drivers of GFP expression, and one of them (F4-678) even had a close grouping of Mef2 and CF2 sites, which likely accounted for strong larval somatic muscle expression. It is unlikely that CF2 could be causing changes in *mhc* levels by affecting an untested enhancer while having no effects on the enhancers we found, but we cannot completely rule out the possibility that we may have separated IFM enhancers away from more distant regulatory elements.

### CF2 may “fine tune” filament stoichiometry

In *mhc* or *Act88F* heterozygotes, there is no upregulation of the remaining wild type copy sufficient to correct the imbalance in filament stoichiometry. This could mean that there is no system for such upregulation, or alternatively, any existing system is not strong enough to reverse that great an imbalance. It is interesting to note that in *Mhc* *[1]* */+* IFM, the myofibrils are ∼ 30% smaller than wild type [2], which could represent a not quite successful attempt by such a putative system to regain proper filament ratios. In contrast, two of the strongest *CF2* mutants (as measured by CF2 protein level) have wild type-like hexagonal arrangements of filaments, in myofibrils that are up to 50% to 60% larger than wild type. While we have examined only one thick filament gene and two thin filament genes, it is likely that expression of other structural genes is also modulated, directly or indirectly. It has been noted that CF2 is not absolutely required for initiation of expression of embryonic muscle genes, but rather it functions to modulate levels of gene expression [13], [14]. CF2 may play a similar role in the control of *Act88F* expression, except as a repressor in the IFM context. As a modulator, a reduction in its function would have a milder effect, at least in the cases of alleles such as *04624* and *R3*, an effect possibly weak enough to allow correction within the capabilities of a potential stoichiometry sensing/maintenance system. The phenotypes of these two mutants could present an opportunity to screen for genes involved in sensing and/or maintaining myofibril size or filament stoichiometry, by providing a sensitized background.

## Materials and Methods

### Fly stocks and genetics

All fly stocks were raised on standard media at 22°C unless otherwise specified. *yw[67c23]* served as the wild**-**type control, *CF2* mutant stocks *CF2[KG05342]* and *CF2[KG08941]* were obtained from the Bloomington Stock Center (University of Indiana, Bloomington IN), *CF2[c04624]* and *CF2[c01640]* from the Harvard Exelixis collection (Harvard Medical School, Boston MA), and UAS-CF2-RNAi (11924-R2) from the National Institute of Genetics (Mishima, Shizuoka, Japan). The *CF2[KG08941-R3]* mutant was generated by P-element excision of the *CF2[KG08941]* stock. The *mhc-GFP* [23] and *UAS-CF2-II[wt]* and *UAS-CF2-II[A40]* strains [18] have been described previously.

### Construction of IFM GAL4 driver lines

A 1.3 kb fragment of *Act88F* upstream region was amplified via PCR using the primers 5′-ggatccaaataaaacgctttgggaatgcc-3′ and 5′-ggatccttcgacattgaggtcgcactc-3′. The fragment was cloned into a GAL4 vector described previously [24] and used to make transgenic strains by the standard methodology. Transformant lines were tested for IFM expression by crossing to UAS-GFP. GFP expression is first observed in the IFM at around 40 hours APF (after pupa formation), and in the upper legs of pharate adults, a pattern reported previously [25]. A chromosome II line, 81B, was recombined with *Act88F-GFP* (fly line from S. Bernstein) to produce line 81B-13. A chromosome III line, 69, was recombined with a *mhc-GFP* (from E. Chen) to produce line 69-5. The mhc-GAL4 driver was made with a 580 bp upstream fragment that first shows IFM expression at about 40 hours APF (see Figs. 6A and B). A mhc-GAL4, mhc-RFP recombinant line was made via standard techniques.

### Immunostaining of adult IFM

Adults were fixed using the high-octane fixation protocol (R. Carthew). IFM were stained with anti-CF2 antibody (1∶1000) and a biotinylated goat anti-rabbit 2° antibody (1∶1000, Vector, Burlingame, CA). Photographs were taken with a Zeiss Axioplan 2 microscope and digital camera using AXIOVISION V3.1 software.

### qPCR

RNA from whole flies of the appropriate genotype was purified using TriZol (Invitrogen, Carlsbad, CA), and treated with RQ1 DNAse (Promega, Madison, WI) to remove any residual genomic DNA. The Superscript II cDNA kit (Invitrogen) was used to make cDNA. All qPCR reactions (total volume of 10 ul) were done in triplicate with the Applied Biosystems (Foster City, CA) SYBR Green Master Mix, 400 ng of cDNA, 50 nM primers, in an Applied Biosystems 7500 Fast Real Time PCR System using the 7500 v2.0.1 software. The primers (exact sequences available upon request) were designed to include flight muscle specific exons when applicable (exon 3 for *Tn I* and exon 11e for *mhc*). Measurement of GAPDH1 RNA levels was used as the reference.

### RT-PCR

RNA and cDNA from freeze-dried thoraces, dissected IFM, or whole flies was prepared as described previously [26]. The sequences of the primers used are available upon request.

### Paraffin sections

Embedding and sectioning of adult thoraces was done as described previously [26]. Photographs were taken as described above with a GFP filter.

### TEM of flight muscles

Indirect flight muscles were prepared from 1 to 2 day old adults for transmission electron microscopy and photographed as described previously [26]. Myofibril sizes were quantified by counting the thick filaments of 13 single myofibrils magnified at 60,000X for each genotype. Differences from wild type were tested for statistical significance with student's t-test.

### Construction of *mhc* and *Act88F* reporters

The 88Factin reporter was made by inserting the 1.3 kb fragment used for construction of the GAL4 driver into a Pelican vector with the GFP region replaced with an RFP construct (Ds-Red from BD Biosciences, San Jose, CA).

A 9-kb region upstream of the *mhc* gene was amplified via PCR to produce a series of overlapping fragments (primer sequences available upon request). These fragments were cloned into the Green Pelican vector and used to create transgenic fly strains via standard techniques. Transgenic animals were monitored under fluorescent light during the larval and pupal stages to determine any muscle expression pattern. Live animals expressing GFP or RFP were photographed with a Leica MZFLIII Stereomicroscope, using ImagePro 6.0.

### Western blotting

Freeze dried IFM or whole thoraces of each genotype were pooled, ground in liquid nitrogen, and resuspended in 50 µl dH_2_O/50 µl Western sample buffer (125 mM Tris 6.8, 6% SDS, 20% glycerol, 0.025% bromophenol blue, 10% β-mercaptoethanol), and heated to 100°C for 10 minutes. Samples were resolved on 8% polyacrylamide gels and transferred to Immobilon-P (Millipore, Billerica, MA) using standard protocols. Membranes were incubated overnight at 4°C with CF2 antibody (a polyclonal that detects all three isoforms) at a dilution of 1∶15,000 or actin antibody (MAB 150 1, Millipore) at a dilution of 1∶1000. Secondary antibody (anti-rabbit-HRP for CF2, anti-mouse-HRP for actin, Pierce, Rockford, IL) was used at a dilution of 1∶4000. Protein bands were visualized using SuperSignal West Femto Maximum Sensitivity Substrate (Pierce).

## References

[1] Barbas JA, Galceran J, Torroja L, Prado A, Ferrús A (1993). Abnormal muscle development in the heldup^3^ mutant of *Drosophila melanogaster* is caused by a splicing defect affecting selected troponin I isoforms.. Mol Cell Biol.

[2] O'Donnell PT, Bernstein SI (1988). Molecular and ultrastructural defects in a *Drosophila* myosin heavy chain mutant: differential effects on muscle function produced by similar thick filament abnormalities.. J Cell Biol.

[3] Beall CJ, Sepanski MA, Fyrberg EA (1989). Genetic dissection of *Drosophila* myofibril formation: effects of actin and myosin heavy chain null alleles.. Genes Dev.

[4] Reedy MC, Beall C (1993). Ultrastructure of developing flight muscle in *Drosophila*. I. Assembly of myofibrils.. Dev Biol.

[5] Cripps RM, Becker KD, Mardahl M, Kronert WA, Hodges D (1994). Transformation of *Drosophila melanogaster* with the wild-type *myosin heavy-chain* gene: rescue of mutant phenotypes and analysis of defects caused by overexpression.. J Cell Biol.

[6] Ranganayakulu G, Zhao B, Dokidis A, Molkentin JD, Olson EN (1995). A series of mutations in the D-MEF2 transcription factor reveal multiple functions in larval and adult myogenesis in *Drosophila*.. Dev Biol.

[7] Arredondo JJ, Ferreres RM, Maroto M, Cripps RM, Marco R (2001). Control of *Drosophila* paramyosin/miniparamyosin gene expression. Differential regulatory mechanisms for muscle-specific transcription.. J Biol Chem.

[8] Marín MC, Rodríguez JR, Ferrús A (2004). Transcription of *Drosophila* troponin I gene is regulated by two conserved, functionally identical, synergistic elements.. Mol Biol Cell.

[9] Lin MH, Nguyen HT, Dybala C, Storti RV (1996). Myocyte-specific enhancer factor 2 acts cooperatively with a muscle activator region to regulate *Drosophila* tropomyosin gene muscle expression.. Proc Natl Acad Sci U S A.

[10] Kelly KK, Meadows SM, Cripps RM (2002). *Drosophila* MEF2 is a direct regulator of Actin57B transcription in cardiac, skeletal, and visceral muscle lineages.. Mech Dev.

[11] Hsu T, Bagni C, Sutherland JD, Kafatos FC (1996). The transcription factor CF2 is a mediator of EGF-R-activated dorsoventral patterning in *Drosophila* oogenesis.. Genes Dev.

[12] Bagni C, Bray S, Gogos JA, Kafatos FC, Hsu T (2002). The *Drosophila* zinc finger transcription factor CF2 is a myogenic marker downstream of MEF2 during muscle development.. Mech Dev.

[13] Tanaka KK, Bryantsev AL, Cripps RM (2008). Myocyte enhancer factor 2 and chorion factor 2 collaborate in activation of the myogenic program in *Drosophila*.. Mol Cell Biol.

[14] García-Zaragoza E, Mas JA, Vivar J, Arredondo JJ, Cervera M (2008). CF2 activity and enhancer integration are required for proper muscle gene expression in *Drosophila*.. Mech Dev.

[15] Hsu T, Gogos JA, Kirsh SA, Kafatos FC (1992). Multiple zinc finger forms resulting from developmentally regulated alternative splicing of a transcription factor gene.. Science.

[16] Gogos JA, Hsu T, Bolton J, Kafatos FC (1992). Sequence discrimination by alternatively spliced isoforms of a DNA binding zinc finger domain.. Science.

[17] Thibault ST, Singer MA, Miyazaki WY, Milash B, Dompe NA (2004). A complementary transposon tool kit for Drosophila melanogaster using P and piggyBac.. Nat Genet.

[18] Mantrova EY, Hsu T (1998). Down regulation of transcription factor CF2 by *Drosophila* Ras/MAP kinase signaling in oogenesis: cytoplasmic retention and degradation.. Genes Dev.

[19] Hess NK, Singer PA, Trinh K, Nikkhoy M, Bernstein SI (2007). Transcriptional regulation of the *Drosophila melanogaster* muscle myosin heavy-chain gene.. Gene Expr Patterns.

[20] Koana T, Hotta Y (1978). Isolation and characterization of flightless mutants in *Drosophila melanogaster*.. J Embryol Exp Morphol.

[21] Barthmaier P, Fyrberg E (1995). Monitoring development and pathology of *Drosophila* indirect flight muscles using green fluorescent protein.. Dev Biol.

[22] Marco-Ferreres R, Arredondo JJ, Fraile B, Cervera M (2005). Overexpression of troponin T in *Drosophila* muscles causes a decrease in the levels of thin-filament proteins.. Biochem J.

[23] Chen EH, Olson EN (2001). Antisocial, an intracellular adaptor protein, is required for myoblast fusion in *Drosophila*.. Dev Cell.

[24] Gajewski K, Choi CY, Kim Y, Schulz RA (2000). Genetically distinct cardial cells within the *Drosophila* heart.. Genesis.

[25] Nongthomba U, Pasalodos-Sanchez S, Clark S, Clayton JD, Sparrow JC (2001). Expression and function of the *Drosophila* ACT88F actin isoform is not restricted to the indirect flight muscles.. J Muscle Res Cell Motil.

[26] Gajewski KM, Wang J, Schulz RA (2006). Calcineurin function is required for myofilament formation and troponin I isoform transition in *Drosophila* indirect flight muscle.. Dev Biol.

